# Level of Agreement Between Plaque Detection with Clinical Assessment and Assessment on Intraoral Scanner

**DOI:** 10.3390/dj12120395

**Published:** 2024-12-05

**Authors:** Grigoria Gkavela, Pia Elisabeth Nørrisgaard, Christos Rahiotis

**Affiliations:** 1 Department of Operative Dentistry, School of Dentistry, National and Kapodistrian University of Athens, 11527 Athens, Greece; grigoriagk@dent.uoa.gr; 2 Senior Clinical Research Manager, 3Shape TRIOS A/S, 1060 Copenhagen, Denmark

**Keywords:** level of agreement, intraoral scanner, plaque, disclosing agent, plaque index

## Abstract

**Background/Objectives:** The aim was to evaluate the agreement between plaque detection with an intraoral scanner system (IOS) and a conventional clinical method and to evaluate the inter-rater reliability for scoring 3D models with and without a disclosing agent. **Methods**: A total of 14 participants were recruited from the Department of Operative Dentistry, School of Dentistry, National and Kapodistrian University of Athens. Participants eligible for inclusion were adults with good general health and a minimum of 20 teeth. Participants were clinically examined with plaque assessment according to the modified Quigley–Hein plaque index before and after using a disclosing agent (GC-Tri Plaque ID-Gel, GC, Europe N.V). Before and after the application of the disclosing agent, all study participants were scanned using the IOS (TRIOS5, 3Shape TRIOS A/S). The clinical examiner and three additional examiners blinded to the clinical examination assessed plaque status on the acquired 3D models with and without disclosing agent using the same index to evaluate the inter-rater agreement. Intraclass coefficient correlation, one sample t-test, and Cronbach’s α for inter-rater reliability were calculated. **Results**: All methods showed moderate to strong correlations (Spearman’s rho ranging from 0.527 to 0.618), and Cronbach’s α ranged from 0.551 to 0.766. **Conclusions:** The level of agreement between conventional clinical registration and registration from 3D models was acceptable overall.

## 1. Introduction

Dental plaque is a bacterial biofilm that accumulates on the tooth surfaces and is directly responsible for caries and periodontal diseases, the most prevalent oral diseases worldwide, affecting people of all ages [1,2]. Usually, it accumulates at the gingival margin and in the interproximal areas. The enamel’s natural, smooth, shiny appearance is lost with the accumulation, and a dull and matt effect is produced [1]. As it builds up, masses of dental plaque become more readily visible to the naked eye. After being undisturbed and for a few days of accumulation, the biofilm matures, creating a risk for dental caries and periodontal disease development [3].

Gingivitis has a high prevalence affecting a significant portion of the population and is prevalent in all ages. Gingivitis is a precursor to periodontal disease, characterized by periodontal pockets, which provide an ideal environment for bacteria to grow and spread the infection, resulting in damage to the underlying bone, i.e., bone loss. If the progression of periodontitis is not halted, it can lead to tooth movement and eventually to tooth loss. Therefore, dental plaque identification and oral hygiene control are crucial for maintaining oral health and preventing disease development [1,4,5].

The detection of dental plaque is challenging. Methods traditionally used for clinical plaque detection and identification are visual inspection, scrubbing the tooth surface with a dental probe, or applying a disclosing agent that colors the plaque [1].

These clinical procedures are usually time-consuming and somewhat subjective. Nowadays, 2D intraoral cameras employ fluorescence to excite the red fluorescence from porphyrins in dental plaque, and their metabolites are also available. They show reliable diagnostic performance and the potential to replace disclosing agents (Quantitative light-induced fluorescence–QLF) [6,7,8,9]. Since the red fluorescence’s intensity depends on the type of microbes and the maturity of the dental plaque, the QLF images could differ significantly, making the diagnosis from the images difficult [9,10].

Many clinical indices have been used to quantify the presence of plaque. Some of those indices used through the years are the Quigley–Hein index (or the modified version by Turesky), the Silness and Loe, the O’Leary, the Mandel, the Fischman, and the Navy. Nevertheless, all the indices introduce subjectivity to the process [8,11]. The modified Quigley–Hein index is used in many digital image studies because of its simplified criteria and its correlation to clinical outcomes such as gingival inflammation [8]. Computerized methods are superior in objectivity, sensitivity, and reliability [11]. Intraoral scanners (IOS) are handheld devices that capture high-resolution 3D images of the teeth and surrounding oral tissues. These images are generated through optical scanning technology, which creates detailed digital impressions. The IOS devices can also record intraoral information as photorealistic color images. IOS can also detect plaque by capturing detailed images of the tooth surfaces, highlighting areas where plaque accumulation is evident. The resulting images can be used for educating patients in oral hygiene and patient comparative clinical plaque evaluation, and it is yet to find out if they could be used in studies. Recent publications indicate that IOS may be suitable for plaque detection and registration [12,13,14,15]. Although all the recent research papers have different methodologies, all seem to agree that plaque scoring with an IOS correlated well with clinical examination. Also, Giese-Graft et al. (2022) conclude that 2D models can be used for plaque monitoring with adequate agreement with clinical recordings [14]. Doi et al. (2021) address the issue by examining different tooth groups, finding the more significant differences in plaque scoring for mandibular anterior teeth, but overall, there is a good agreement [12]. The studies used a disclosing agent, and the level of agreement between oral observation with and without a disclosing agent is well established [12,13,14].

The 3D models IOS produces can be shared with patients, helping them understand the extent and location of plaque buildup. This visual evidence can motivate better oral hygiene practices. Additionally, digital records are easily stored and accessed, facilitating seamless communication between different healthcare providers if needed. They can also support monitoring and evaluating oral hygiene educational instructions for the patient. Finally, the high-resolution images allow dentists to thoroughly assess a patient’s oral hygiene from a distance. However, more information must be provided on how the inter-rater agreement among different examiners evaluates the 3D IOS models.

This study evaluates the agreement between an IOS and a conventional clinical visual method with a disclosing agent for dental plaque detection and registration. The aim was to evaluate the agreement between the IOS and a conventional clinical method, the agreement between 3D images with and without a disclosing agent, and the inter-rater reliability for scoring 3D models with and without a disclosing agent.

## 2. Materials and Methods

### 2.1. Study Design and Participants

The study group was a convenience sample of 14 participants (eight female, six male) with a mean (±SD) age of 37.5 ± 1.2 years, recruited via oral announcements from the Department of Operative Dentistry, School of Dentistry, National and Kapodistrian University of Athens. The study was approved by the Ethics and Research Committee of the School of Dentistry (527/22 September 2022) of the National and Kapodistrian University of Athens. The study was conducted from October 2022 to March 2023.

All participants were informed about the aims and methodology of the study, and written consent was obtained.

Participants eligible for inclusion were adults with good general health and a minimum of 20 permanent teeth that did not have orthodontic appliances or fixed prosthodontic restorations. Teeth with active carious lesions or with resin/amalgam restorations were included in the study. Exclusion criteria were less than twenty teeth in the mouth, medical conditions that made the registration difficult or impossible, age under 18, and inability to obtain informed consent.

### 2.2. Clinical Procedures

One examiner conducted all the clinical procedures. The examiner was a dental professional calibrated for clinical plaque registration before the study and trained in using the intraoral scanner (TRIOS5, 3Shape TRIOS A/S, Copenhagen, Denmark).

At first, scanning the participant’s arches was conducted using the scanner with Unite application software. A comprehensive clinical examination and plaque registration were performed with visual observation and not using a periodontal probe (as the plaque accumulation needed to be intact for the following steps). The index used for the registration was the modified Quigley–Hein plaque index [16] (Table 1). A full-mouth plaque assessment was conducted in this study, with each tooth evaluated buccally and lingually. Subsequently, a disclosing agent was applied to all tooth surfaces (GC-Tri Plaque ID-Gel, GC Corporation, Tokyo, Japan). The agent was applied with a plier and a cotton pellet, and the participant was instructed to rinse for 5 s with gentle moves and then spit.

Following the application of the disclosing agent, all study participants were scanned with the intraoral scanner once again. Afterward, a thorough professional cleaning was performed on all tooth surfaces, and the stains and the disclosing agent were removed by polishing using a polishing paste (Detartrine polishing paste, Septodont, Toronto, ON, Canada) and a rubber cup.

**Figure 1:**
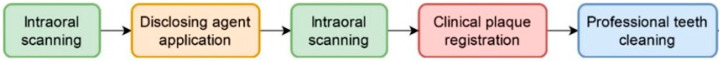
An overview of the methodology of the study is provided.

Finally, oral hygiene instructions were demonstrated to the subject. Figure 1 presents an overview of the study’s workflow.

After all data were obtained, the examiner (Examiner 1) and three additional dental professionals (Examiners 2, 3, 4) blinded to the clinical examination assessed plaque status on the acquired 3D models with and without disclosing agent using the same index to evaluate the inter-rater agreement. The 3D models were assessed using the Unite application software. In this viewer, the 3D models can be rotated in all three directions, so all areas of the dental arch are well displayed. All the additional examiners had received the same clinical plaque registration training before this study. However, the examiner who completed the initial clinical examination and Examiner 4 have the most clinical experience (Examiner 1 is a restorative dentist, and Examiner 4 is a dental hygienist and were both trained in using indexes for many years). In contrast, the other two were not as experienced in plaque detection.

### 2.3. Statistical Analysis

The sample size calculation was based on preliminary tests with five individuals (data not included in the present study) and was performed using the G* Power version 3.1.9.7. As there was no agreed value for a clinically relevant difference in the agreement of the measured *p* % values, a permitted difference of 10 *p* % units was defined as clinically relevant for the sample size calculation. The sample size was calculated to be at least 12 participants. Statistical analyses were performed using the IBM SPSS 26.0 (IBM Corp., Armonk, NY, USA) software and included descriptive statistics and the calculation of interclass coefficient correlation. The data showed significant deviation from the Gaussian distribution (Kolmogorov–Smirnov test), so non-parametric procedures were used. Spearman correlation was used to examine whether the plaque registration on 3D models and plaque recording on 3D models with disclosing agent correlated with the clinical plaque registration. Also, one sample t-test was conducted for inter-rater reliability. Bland–Altman analyses were performed to determine the extent to which the measurements obtained with clinical examination agreed with those of 3D models examination and 3D models with disclosing agent. For the inter-examiner reliability of all four examiners, Cronbach’s α was calculated. Intra-examiner reliability was assessed using the Intraclass Correlation Coefficient (ICC) for repeated measures by the primary examiner. The analysis used pooled data and site-specific comparisons to ensure a comprehensive evaluation. Pooled data provided an overall assessment of agreement between the methods, while site-specific comparisons allowed for detailed analysis of differences across various tooth groups, such as anterior versus posterior teeth and mandibular versus maxillary teeth. This dual approach ensured a nuanced understanding of plaque detection performance across different oral regions. The level of statistical significance was set at *p* ≤ 0.05.

## 3. Results

**Figure 2:**
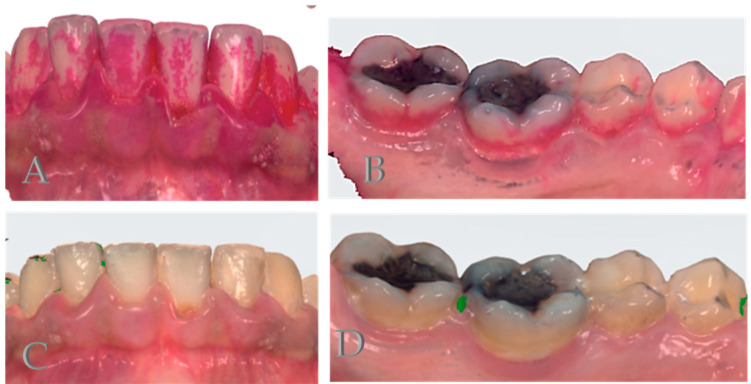
Images of 3D models with (**C**,**D**) and without (**A**,**B**) disclosing agents in which it is presented how different sites (lower incisors- buccal on (**A**,**C**) and lower molars and premolars-lingual on (**B**,**D**)) can be presented in a 3D image.

Images of 3D models with and without disclosing agents are shown in Figure 2. All three ways of scoring plaque (clinical examination, 3D models, and 3D models with disclosing agent) showed moderate to strong correlations (Spearman’s rho ranging from 0.527 to 0.618) with *p* < 0.001 (Table 2). When the scoring techniques were correlated per tooth group type (upper front, lower front, upper molars and premolars, lower molars, and premolars), the correlations were also good (*p* < 0.001), with the lowest correlation for the upper front when comparing 3D models with or without disclosing agent (0.450) and the highest correlation in for the upper front when comparing 3D models with disclosing agent and clinical examination (0.763) (Table 3).

**Figure 3:**
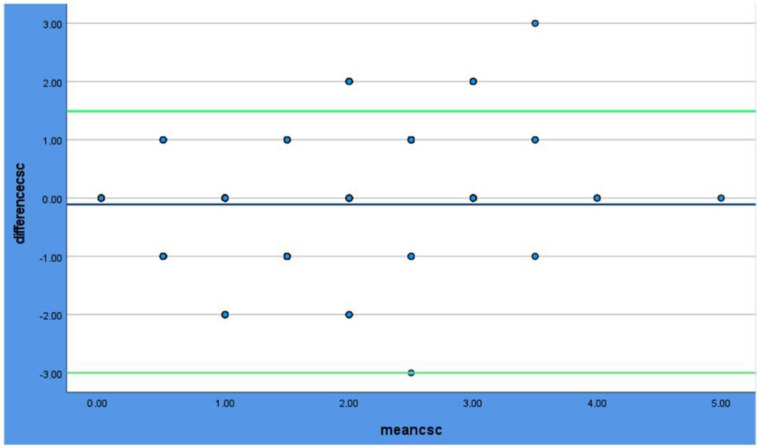
BlandAltman plot for Clinical Vs. Scanner. The blue line shows the mean value. The Green lines show the limits of the agreement (±1.96). The dots are the measurements.

**Figure 4:**
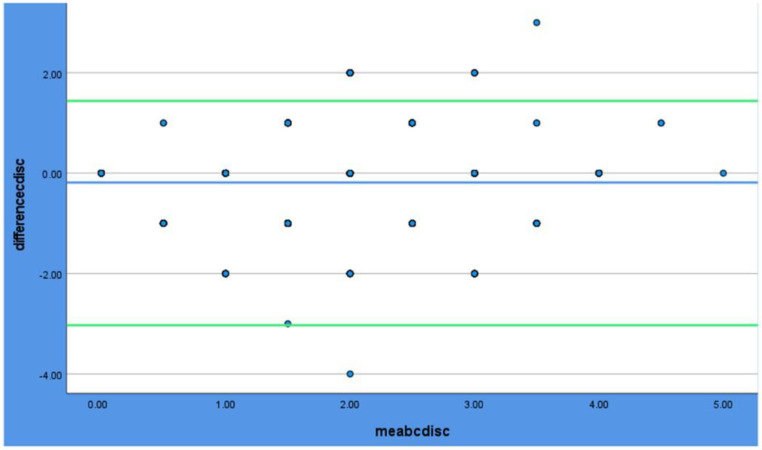
Bland–Altman plot for Clinical Vs. Disclosing. The blue line shows the mean value. The Green lines show the limits of the agreement (±1.96). The dots are the measurements.

**Figure 5:**
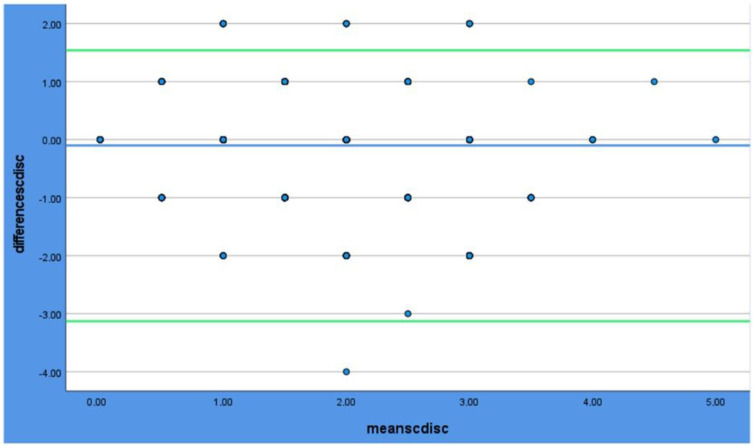
Bland–Altman plot for Scanner Vs. Disclosing. The blue line shows the mean value. The Green lines show the limits of the agreement (±1.96). The dots are the measurements.

One sample *t*-test was conducted prior to the Bland–Altman plots to determine whether there is a significant bias between the methods; the results are shown in Table 4. In the Bland–Altman plots (Figure 3, Figure 4 and Figure 5), all the methods of scoring plaque show excellent agreement, as the majority of the dots are between the levels of agreement (green lines). 

Cronbach’s α for the interrater reliability ranged from 0.551 to 0.766 (Table 5). The correlation was stronger between Examiner 1 and Examiner 4.

The ICC value was calculated to be 0.82, indicating good reliability and consistency in the examiner’s plaque detection assessments over time. This strong reliability underscores the robustness of the examiner’s evaluations and the reproducibility of the clinical methodology utilized in this study.

## 4. Discussion

This is the first study to investigate the agreement between clinical plaque scores and plaque scores on 3D models with and without a disclosing agent, in addition to the agreement between several examiners. Spearman’s rho values less than 0.5 indicate poor reliability, between 0.5 and 0.75 indicate moderate agreement, values between 0.75 and 0.9 indicate good reliability, and greater than 0.90 indicate excellent reliability [17]. All three methods of scoring plaque (clinical examination, 3D models, or 3D models with disclosing agent) correlated moderate to strong. In a scoping review in 2023, it was stated that a moderate to strong positive correlation was found between the digital methods and the clinical examination for plaque detection, with comparable results for 2D and 3D image-based systems [18]. Even though we found moderate to strong correlations for all three methods of scoring plaque (clinical examination, 3D models, and 3D models with disclosing agent), the correlation between the clinical examination and the 3D models with disclosing agent was slightly higher than for the 3D models without disclosing agent, which indicates that examiners can easier detect plaque if it is colored.

In a study of QLF imaging, a strong correlation was found between all disclosed plaque scores and the clinical scores in a study using QLF images and a disclosing agent like the one used in the present study. In our study, the correlation was higher when a disclosing agent was used. The latter is likely because the disclosing agent gives additive information on the 3D model closest to the clinical examination. Indeed, a recent study showed a strong correlation between clinical registrations on disclosed and undisclosed 3D models [13], which aligns with our results.

Discrepancies between agreements in different groups of tooth types do not deflect from bad scanning quality but from different types of plaque accumulating at different sites. In a study in 2021, the difference between plaque detected on 3D models with and without disclosing agents was found to be more significant for mandibular anterior teeth [12]. Also, in a study of QLF imaging, it was concluded that the reliability of registering images with a disclosing agent is site-dependent [9]. In another recent study, a site-specific comparison revealed that the scores obtained using the intraoral scanner were significantly higher in the posterior region in the maxilla and mandible, possibly due to difficulty in direct vision [17]. We also reported the same pattern, which can be attributed to the scanner providing better images in sites where optical observation is compromised (e.g., posterior teeth). Also, those sites are typically sites that are prone to plaque accumulation, mostly because of poor access to oral hygiene methods. This could mean that the examiners might have a better agreement for higher scores on the index but that was not statistically tested in this study.

The intra-class correlation coefficient (ICC) provides a single measure of the extent of agreement and the Bland–Altman plots provide a quantitative estimate of how closely the values from the two measurements lie. The Bland–Altman plot can show both the magnitude of bias and 95% limits of agreement between the two methods. In this study, most of the dots were between the lines for all the scoring plaques, so we can conclude that there was a very high level of agreement between different methods for all techniques. However, the one-sample t-test for 3D models with or without disclosing agent reveals that the upper bound for the correlation of clinical examination and the 3D models was −0.0416, and for the correlation of clinical examination and 3D models with disclosing agent was −0.1368. Let us consider that confidence intervals provide a way to express uncertainty and account for variability in our estimates. This indicates that for the 3D models with disclosing agents, the upper boundary of the interval is further from the central estimate. Conclusively, there is more significant uncertainty or variability in that direction.

The inter-rater reliability in this study ranged from 0.551 to 0.766. The interpretation of the inter-rater reliability estimates should be 0.70 to be acceptable, 0.80 to be good, and 0.90 to be excellent [19]. Only inter-rater reliability between examiners #1 and #4 and #2 and #4 was above 0.70 and can be characterized as acceptable, while the reliability between examiners #3 and #4, #1 and #2, and #2 and #3 did not reach the acceptable threshold, but they were close to that and could be characterized as “close to acceptable”. These results could be attributed to the fact that Examiners 1 and 4 were specialists and more experienced than Examiners 2 and 3. So that might mean that with better-trained examiners the results would be “acceptable”. Previous reports showed that intra- and inter-rater reliability for digital measures was good/excellent when assessed [18]. This finding allows us to comment that better training of the examiners (for the clinical use of this plaque registration system) would increase the inter-rater reliability. Methods of increasing interrater reliability that could mean a clinical improvement of the plaque registration system could be better and ongoing training of the examiners, with regular calibration sessions and minimizing the complexity of the task setting criteria with more clarity [20].

Digitized plaque detection systems were reported to be reliable and valid tools for dental plaque monitoring and oral hygiene assessment [18]. Also, in a study of 2023, where different types of plaque registration were evaluated, 3D imaging was found to have an advantage, as it allows image enlargement and rotation at all sites [21].

As previously stated, it is well-known that using clinical indices to register plaque detection introduces subjective bias [11]. Another restriction of using the indices is that, in many cases, the identical scores on teeth of equal size may be due to different amounts of plaque (different thicknesses). The opposite may also be true: using tooth segments to obtain scores effectively disregards tooth size. This means that the same amount of plaque may yield different scores on different-sized teeth (i.e., central and lateral incisors) [11]. 

Also, two-dimensional images have some disadvantages. One is that lingual surfaces and posterior teeth are assessed with mirrors and difficulty. The difficulty of acquiring images on certain surfaces remains even for intraoral cameras. So, curvature plays a part in imaging. In addition, interproximal sites cannot be reached as well as with clinical examination [11]. One possible advantage of 3D imaging for plaque registration is that it also records plaque accumulations on gums, which could be beneficial [11].

A significant limitation of plaque registration through digital 3D models could be that it is challenging to differentiate between plaque and calculus. This was the reason that in this study, the ICC was tested between plaque registration through digital 3D models with and without a disclosing agent, and the results showed a moderate to strong correlation. This result indicates that even though it could be difficult, it is safe on a clinical note to differentiate between calculus and plaque in the 3D images, depending, of course, on the examiner’s experience.

Not differentiating between plaque and calculus can introduce several biases. Plaque and calculus have different microbial compositions. Plaque consists of active microbial communities, while calculus is a mineralized form of plaque that preserves microbial DNA and proteins over time. Plaque represents an early stage of biofilm development, whereas calculus is a more mature, calcified state. This distinction is crucial for understanding the dynamics of biofilm formation and the associated microbial activities. Plaque is directly responsible for oral pathologies such as caries and periodontal disease, while calculus is more of a historical record of past microbial activity, although, primarily due to its porous texture, calculus could be more easily found covered with plaque [2].

The limitation of this study is that only some participants were included. The sample size for this study was determined using G* Power 3.1 software. While this sample size meets the statistical requirements for a pilot study, we acknowledge its limitations in terms of generalizability. Small sample sizes inherently reduce the ability to extrapolate findings to larger populations and may not capture variability in plaque patterns across diverse demographics. Despite this limitation, the study provides valuable preliminary insights into the agreement between plaque detection methods. Future research should aim to replicate these findings in larger, more diverse cohorts to confirm the results and enhance their applicability to broader populations. While the study utilized a convenience sample recruited via oral announcements, which may introduce potential selection bias, strict inclusion and exclusion criteria were employed to minimize variability and enhance the reliability of the findings. The eligibility criteria ensured that participants represented a population with sufficient dentition and oral health status to assess plaque detection methods accurately. However, the recruitment method limits the generalizability of the results. Even though we registered plaque per surface in this study and had 769 surfaces included in the cross-examination, the conclusions should be taken cautiously. Only participants with very few stains were included, so we could not assess how stains could impact the outcome. The Quigley–Hein index used in the present study is generally considered a subjective index for plaque scoring. Nevertheless, some subjectivity issues arise as different examiners could interpret plaque coverage differently, especially when not using a periodontal probe for the measure. Also, with the type of index used, it is impossible to differentiate calculus from plaque and assess the differences from the disclosing agent used, which identifies new, mature, and acid-producing biofilms in three colors. Additionally, this index focuses on selected surfaces, which may not represent plaque distribution across all dental surfaces. As a result, variations in plaque accumulation on unexamined surfaces may be overlooked, potentially leading to an incomplete evaluation of oral hygiene or treatment efficacy. However, in a 2022 study that compared two different 3D plaque registration methods, an intraoral camera and an IOS, the registrations obtained from the intraoral camera and the intraoral scan revealed a similar result [14]. In that specific study, there was a tendency for higher scores on both 2D and 3D images compared to the clinical examination, which was explained by the fact that teeth on the images were enlarged compared to the clinical situation, the illumination of the areas under study was usually very good compared to the clinical situation. There were no clinical-related factors [14].

Future studies with more extensive, more diverse, and randomized participant pools are recommended to validate these findings and enhance their applicability to broader populations. Further studies on a larger sample should be conducted. Differences in the methodology of the published papers (e.g., the indices used, the disclosing agent applied, or even the type of intraoral scanner used) could influence the results.

## 5. Conclusions

The overall level of agreement between conventional clinical registration and registration from 3D models was acceptable. The results suggest that the IOS could help assess oral hygiene status and aid in accurately evaluating dental plaque, especially in areas that cannot be viewed directly and require dental mirrors. The clinical experience influences the inter-rater agreement. Further studies on a larger sample would be beneficial.

## Figures and Tables

**Table 1:** The Turesky modified Quigley–Hein plaque index scores and description.

Score	Description
0	No plaque at the cervical margin
1	Separate flecks of plaque at the cervical margin of the tooth
2	A thin continuous band of plaque (≤1 mm) at the cervical margin of the tooth
3	A band of plaque wider than 1 mm but covering less than one-third of the crown of the tooth
4	Plaque covering at least one-third but less than two-thirds of the crown of the tooth
5	Plaque covering two-thirds or more of the crown of the tooth

**Table 2:** Correlations * between the three methods of scoring plaque (clinical examination, 3D models, and 3D models with disclosing agent).

		Clinical	3D Models	3D Models + Disclosing
Clinical	Correlation Coefficient	1.000	0.573	0.618
	*p*-value		<0.001	<0.001
3D models	Correlation Coefficient	0.573	1.000	0.527
	*p*-value	<0.001		<0.001
3D models with disclosing	Correlation Coefficient	0.618	0.527	1.000
	*p*-value	<0.001	<0.001	

* Spearman’s rho.

**Table 3:** Correlations * between the methods of scoring plaque (clinical examination, 3D models, and 3D models with disclosing agent) per tooth group type.

	Clinical Vs. 3D Models	Clinical Vs. 3D Models with Disclosing Agent	3D models vs. 3D Models with Disclosing Agent
Upper molars and premolars	0.563	0.568	0.647
Upper front	0.490	0.763	0.450
Lower molars and premolars	0.595	0.746	0.527
Lower front	0.616	0.543	0.526

* Spearman’s rho.

**Table 4:** One sample *t*-test for inter-rater reliability.

		One-Sided *p*		Two-Sided *p*		95% Confidence Interval of the Difference	
			Df		Mean Difference	Lower	Upper
3D models	−3.204	<0.001	0.649	0.001	−0.10753	−0.1734	−0.0416
3D models with disclosing agent	−6.113	<0 001	0.650	<0.001	−0.20154	−0.2663	−0.1368

**Table 5:** Correlations * for the inter-rater reliability of plaque scored on 3D models with disclosing agent.

	Examiner 1	Examiner 2	Examiner 3	Examiner 4
Examiner 1	1.000	0.664	0.551	0.766
Examiner 2	0.664	1.000	0.681	0.741
Examiner 3	0.551	0.681	1.000	0.648
Examiner 4	0.766	0.741	0.648	1.000

* Cronbach’s α.

## Data Availability

The data presented in this study are available on request from the corresponding author due to privacy and legal reasons.
